# Beyond the clot: diagnostic yield, biomarker performance, and the landscape of alternative diagnoses in emergency CT pulmonary angiography

**DOI:** 10.1007/s10140-026-02494-y

**Published:** 2026-05-30

**Authors:** Francesco Lionetti, Federica Castelli, Giovanni Scavone, Giacomo Tamburino, Salvatore Seminatore, Daniele Carmelo Caltabiano, Luca Mammino, Antonio Basile, Maria Vittoria Raciti, Gianluca Galvano

**Affiliations:** 1 Radiology Unit 1, Department of Medical Surgical Sciences and Advanced Technologies “GF Ingrassia”, University Hospital Policlinico “G. Rodolico-San Marco”, University of Catania, 95123 Catania, Italy; 2 Department of Diagnostic Radiology, Neuroradiology and Interventional Radiology, ARNAS ’Garibaldi Centro’ Hospital, Catania, Italy

**Keywords:** Pulmonary Embolism, Computed Tomography Pulmonary Angiography (CTPA), Emergency Department (ED), Diagnostic accuracy, Radiation exposure

## Abstract

**Background:**

Computed Tomography Pulmonary Angiography (CTPA) is the gold standard for diagnosing acute Pulmonary Embolism (PE). However, overlapping symptoms and reliance on non-specific biomarkers often lead to over-testing. This study provides a comprehensive analysis of CTPA utilization, evaluating diagnostic yield, biomarker performance, and the landscape of alternative thoracic diagnoses with a special focus on radioprotection.

**Methods:**

A retrospective, single-centre study of 659 patients evaluated in the Emergency Department (ED) between January 2023 and November 2025. Data were extensively stratified by patient age, presenting symptoms, D-dimer thresholds, and radiological outcomes.

**Results:**

The global diagnostic yield was 20.9% (138/659). Advanced age correlated linearly with PE prevalence (25.2% in patients > 70 years) but inversely with D-dimer specificity, resulting in a 42% false-positive biomarker rate in the geriatric cohort. Dyspnea was the most common presenting symptom (62%) but demonstrated limited diagnostic specificity for PE. Crucially, in the 521 PE-negative scans, CTPA provided clinically relevant alternative diagnoses in 72.0% of cases (375/521), primarily infectious consolidations and heart failure.

**Conclusion:**

While clinical triage remains within optimal international benchmarks, the standard diagnostic algorithm is heavily confounded by patient age and inflammatory "PE mimics." Integrating age-adjusted D-dimer thresholds and structured pre-test probability scoring is imperative to optimize resource allocation and mitigate unnecessary radiation exposure while preserving diagnostic safety (Righini in JAMA 311:1117–1124, 2014;Konstantinides in Eur Heart J 41:543–603, 2020;).

**Graphical Abstract:**

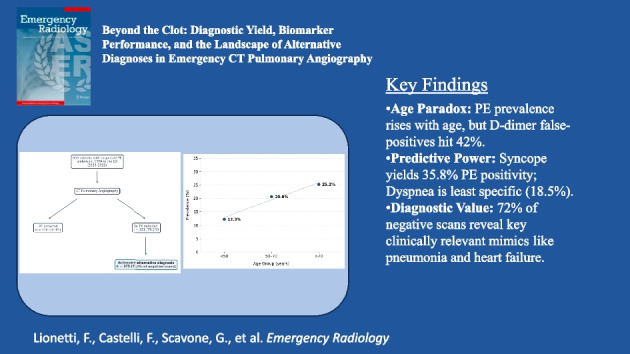

## Introduction

Acute Pulmonary Embolism (PE) is a potentially life-threatening cardiovascular emergency that requires rapid and accurate diagnostic assessment. Computed Tomography Pulmonary Angiography (CTPA) has become the reference imaging modality for suspected pulmonary embolism, owing to its high diagnostic accuracy and its ability to directly visualize intraluminal filling defects within the pulmonary arterial tree [1]. Nevertheless, the diagnostic pathway for suspected PE remains complex because clinical presentation is frequently non-specific and overlaps with a wide spectrum of cardiopulmonary diseases.

Acute PE results from obstruction of the pulmonary arterial tree, leading to increased pulmonary vascular resistance and potential right ventricular overload in severe cases [2].

Furthermore, the classic symptomatic triad of PE—dyspnoea, pleuritic chest pain, and haemoptysis—is notoriously non-specific, shared by a vast array of cardiopulmonary emergencies. The biochemical screening tool, D-dimer, is highly sensitive but lacks specificity, particularly in the presence of systemic inflammation, malignancy, or advanced age [3].

The large multicentre management study by van Belle et al. [4] demonstrated that integrating clinical decision rules (such as the Wells score) with D-dimer testing and selective CT imaging safely excluded pulmonary embolism in a substantial proportion of patients, while maintaining extremely low rates of missed thromboembolic events during follow-up. These findings established the modern stepwise diagnostic strategy for suspected PE and highlighted that imaging should ideally follow a pre-test probability assessment rather than being used as a first-line investigation.

The primary metric for assessing imaging appropriateness is the "diagnostic yield"—the percentage of CT scans positive for thromboembolic disease. While actual adherence to clinical decision rules can be difficult to measure retrospectively, yields between the 15–25% threshold indicate an acceptable balance between diagnostic safety and the avoidance of excessive imaging [5]. Consequently, optimizing CTPA utilization provides a downstream radioprotection benefit, upholding the ALARA (As Low As Reasonably Achievable) principle even when patient-specific dosimetric data is not routinely calculated [6].

This study aims to move beyond a simple binary analysis of "positive versus negative" scans. By comprehensively stratifying a three-year dataset (2023–2025) of 659 patients, we seek to.

1. evaluate the true predictive power of clinical symptoms and D-dimer assays across different age demographics;
2. map the longitudinal trends of diagnostic yield;
3. systematically categorize the "PE mimics"—the alternative pathologies identified when a scan is negative for embolism.

## Materials and methods

### Study design and setting

We conducted a retrospective, cross-sectional diagnostic performance assessment study at a tertiary care centre, analysing all CTPA scans ordered by ED physicians for the clinical suspicion of acute PE over a 35-month period (January 1, 2023, to November 30, 2025). The study protocol was approved by the institutional ethics committee, and the requirement for informed consent was waived due to the retrospective design.

We included consecutive adult patients (≥ 18 years) who underwent CTPA in the ED for suspected PE. The study cohort comprised 659 unique patient encounters. To ensure the analysis of independent clinical events, repeat CTPAs performed on the same patient within a 30-day period were excluded. Pregnant patients were also excluded due to the use of different diagnostic algorithms and imaging protocols. Patients with incomplete essential data (e.g., missing CT reports) were excluded.

### Data extraction and cohort stratification

Data were systematically extracted from six semestral hospital radiological registries. The dataset was rigorously stratified across multiple clinical dimensions:

- Demographics: Patients were stratified into three age cohorts to assess physiological risk variations: Young (< 50 years), Middle-aged (50–70 years), and Geriatric (> 70 years).
- Clinical Presentation: Primary presenting symptoms were clustered into distinct phenotypic groups: Dyspnea, Chest Pain, Syncope/Hemodynamic Instability, and Other/Incidental.
- Biomarker Data: Standard D-dimer assays (quantitative FEA assay, standard institutional positive cutoff ≥ 500 ng/mL) were recorded. Where quantitative values were available, they were mapped against radiological outcomes to evaluate threshold efficacy.

All examinations were performed using multidetector CT scanners (64-slice or higher). To optimize radioprotection, protocols included automated tube current modulation (e.g., CARE Dose4D) and low-kVp settings (100–120 kVp) where appropriate. Institutional protocols are regularly audited against national Diagnostic Reference Levels (DRLs) to ensure dose optimization.

### Radiological evaluation and definitions

CTPA reports were evaluated by board-certified radiologists.

- Positive for PE: Defined as a definitive filling defect within the pulmonary arterial tree (main, lobar, segmental, or subsegmental).
- To address the clinical utility of CTPA beyond pulmonary embolism detection, we evaluated the prevalence of PE mimics and alternative findings. Alternative diagnoses were strictly defined as clinically actionable thoracic findings that could potentially explain the patient’s acute presentation (e.g., pneumonia, pleural effusion, malignancy, mechanical causes, rib fractures, and aortic dissection). Incidental findings not related to the acute symptoms were excluded from this count to ensure a conservative and clinically relevant estimate of the diagnostic yield.

### Statistical analysis

Diagnostic yield was calculated as the proportion of PE-positive scans over the total number of scans. Descriptive statistics, including means, medians, and frequency distributions, were utilized to characterize the cohort.

Data were analysed using professional statistical software. Continuous variables, such as patient age and D-dimer levels, were assessed for normality using the Kolmogorov–Smirnov test. Since D-dimer values exhibited a non-normal (skewed) distribution, they are reported as medians with interquartile ranges (IQR), and comparisons between PE-positive and PE-negative groups were performed using the Mann–Whitney U test.

Categorical variables (symptoms, age cohorts, and diagnostic outcomes) were expressed as absolute frequencies and percentages. Differences between groups were evaluated using the Pearson Chi-square test or Fisher’s exact test where appropriate. To quantify the association between specific symptoms and a positive PE diagnosis, Odds Ratios (OR) with 95% Confidence Intervals (CI) were calculated. (Table 1)All statistical analyses were performed using a dedicated statistical software package (SPSS version 25). Two-tailed tests were used throughout the analysis.

**Table 1:** Diagnostic yield of CTPA and association with temporal, demographic, and clinical variables

*Variable*	*Category*	*Patients (n)*	*PE positive n (%)*	*Odds Ratio (95% CI)*	*p-value*
Study year	2025	151	21 (13.9)	1.00	—
	2024	214	49 (22.9)	1.78 (1.05–3.01)	0.021
	2023	294	68 (23.1)	1.81 (1.10–2.95)	0.015
Age group	< 50 years	114	14 (12.3)	1.00	—
	50–70 years	248	51 (20.6)	1.83 (1.01–3.32)	0.046
	> 70 years	290	73 (25.2)	2.34 (1.29–4.21)	0.004
Primary symptom	Dyspnea	408	75 (18.4)	1.00	—
	Chest pain	145	35 (24.1)	1.40 (0.88–2.22)	0.151
	Syncope	53	19 (35.8)	2.45 (1.35–4.45)	0.003
	Other	53	9 (17.0)	0.91 (0.42–1.95)	0.812

Odds ratios were calculated using the first category of each variable as the reference group. D-dimer levels were measured using a standard quantitative assay, expressed in ng/mL FEU. The institutional positive cutoff was > 500 ng/mL for all patients, as age-adjusted thresholds were not routinely implemented during the study period. A *p*-value < 0.05 was considered statistically significant for all tests.

## Results

**Fig. 1:**
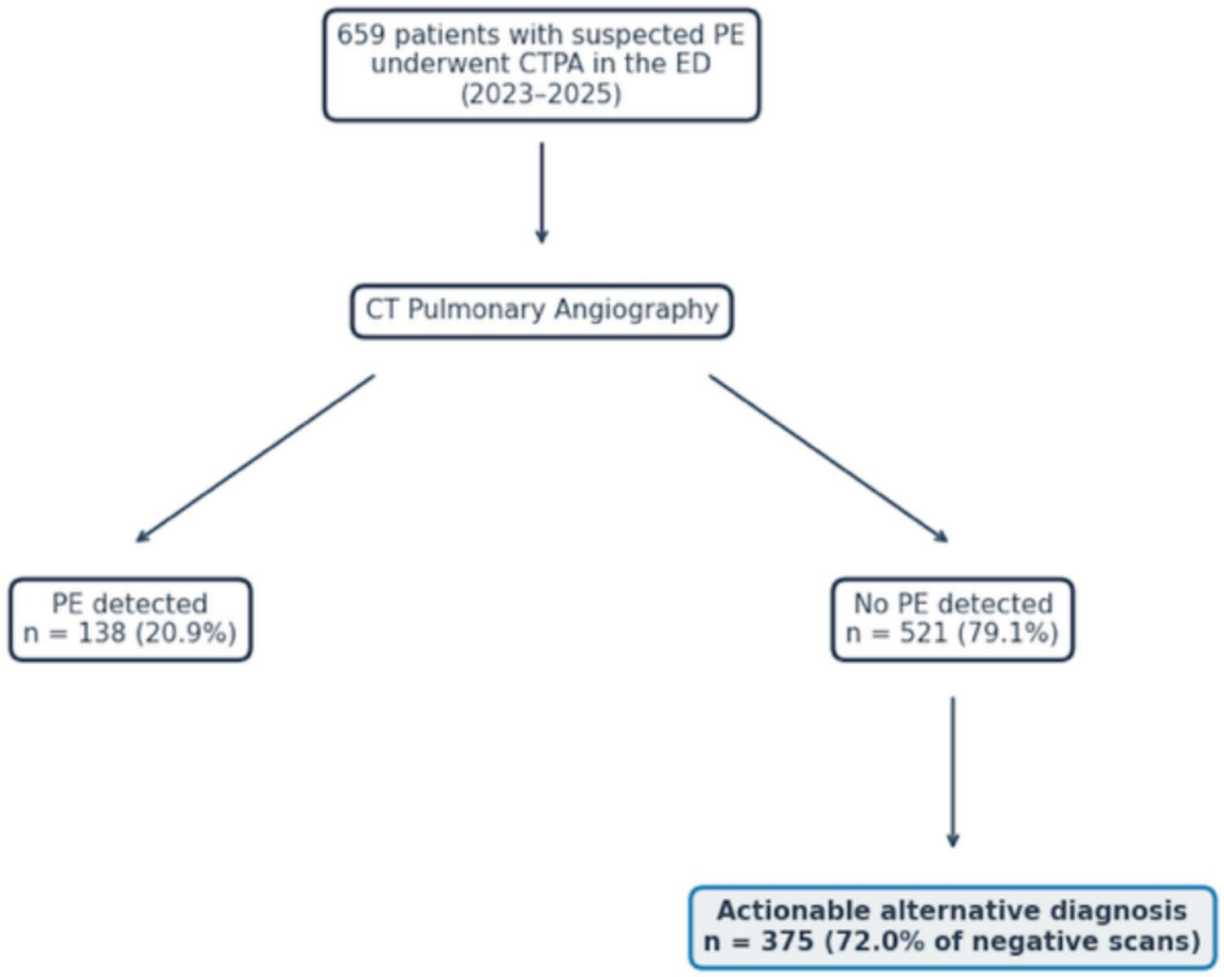
Diagnostic flow of 659 patients undergoing CTPA for suspected pulmonary embolism (ED, 2023–2025)

Pulmonary embolism was confirmed in 138 of 659 patients (20.9%). The diagnostic workflow and outcome distribution are summarized in Fig. 1.

### Overall diagnostic yield and age-stratified diagnostic efficacy

**Fig. 2:**
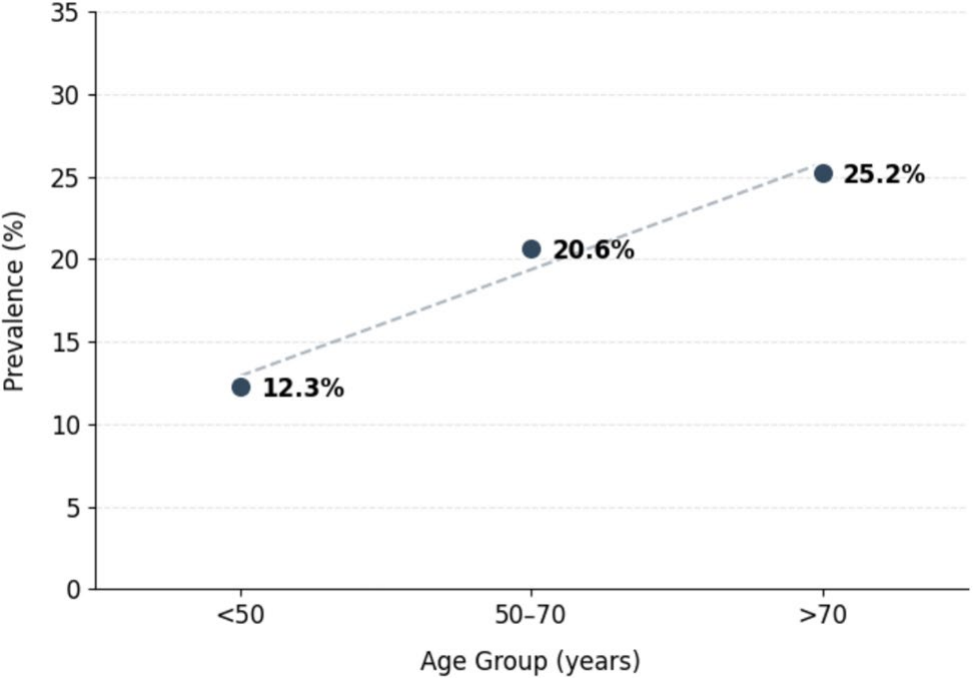
Prevalence of pulmonary embolism detected by CTPA according to patient age group

The overall diagnostic yield was 20.9% (138/659) (95% CI: 17.8%–24.1%). Age proved to be the most significant variable altering diagnostic outcomes (Table 2 and Fig. 2.) Age data was available for 652 out of the 659 patients. The 7 patients with missing demographic data were excluded from the age-stratified sub-analyses but retained in the overall cohort assessments.

- Young Cohort (< 50 years, n = 114): Demonstrated the lowest PE prevalence (12.3%). Negative scans in this group were predominantly strictly normal or showed minor pleuritic changes.
- Middle-aged Cohort (50–70 years, n = 248): Yielded a 20.6% positivity rate.
- Geriatric Cohort (> 70 years, n = 290): Exhibited the highest PE prevalence (25.2%). However, this cohort also presented the highest density of complex, overlapping alternative diagnoses.

**Table 2:** Age-stratified prevalence of pulmonary embolism and D-dimer false-positivity rates

Age Cohort	Total Scans	PE Positive (%)	D-Dimer Positives*
Young (< 50y)	114	12.3%	Low (< 15%)
Middle-Aged (50-70y)	248	20.6%	Moderate (25%)
Geriatric (> 70y)	290	25.2%	High (42%)

*Defined as D-dimer > cutoff but CTPA negative

### Symptomatology and the "Symptom-Diagnosis Gap"

The correlation between pre-test clinical symptoms and post-test radiological confirmation (Table 3) revealed a pronounced "Symptom-Diagnosis Gap”:

- Dyspnea (62% of requests): While overwhelmingly the most common trigger for a CTPA, dyspnea was the least specific symptom. Only 18.5% of isolated dyspnea cases were confirmed as PE; the vast majority were diagnosed with respiratory infections or fluid overload.
- Chest Pain (22%): Demonstrated a moderate predictive value, frequently correlating with distal (segmental/subsegmental) emboli resulting in pulmonary infarction.
- Syncope/Hemodynamic Instability (8%): Carried the highest positive predictive value (> 35%), almost exclusively correlating with massive, central pulmonary emboli causing right ventricular strain.

**Table 3:** Correlation between primary clinical presentation, PE positivity rates, and most frequent alternative diagnoses

*Primary Symptom*	*Frequency*	*PE Positivity Rate*	*Most Common Alternative*
Dyspnea	62%	18.5%	Pneumonia/Heart Failure
Chest Pain	22%	24.1%	Pleurisy/Rib Trauma
Syncope/Hemodynamic Instability	8%	35.8%	Central/Massive PE
Other	8%	9.2%	Neoplasia/Atelectasis

### Biomarker discrepancies: the D-dimer paradox

Analysis of quantitative D-dimer values highlighted severe limitations in its specificity, a phenomenon reflecting the well-documented age-related decline in D-dimer specificity. D-dimer testing prior to CTPA was performed in 88% (580/659) of the cohort.

- True Positives: Patients with confirmed PE exhibited markedly elevated D-dimer levels (median > 7,800 ng/mL).
- False Positives: A substantial proportion of geriatric patients (> 70 years) without PE (42%) demonstrated markedly elevated D-dimer levels (> 2,500 ng/mL), rendering the standard 500 ng/mL cutoff clinically obsolete in this demographic.

### The spectrum of alternative pathologies (PE Mimics)

**Fig. 3:**
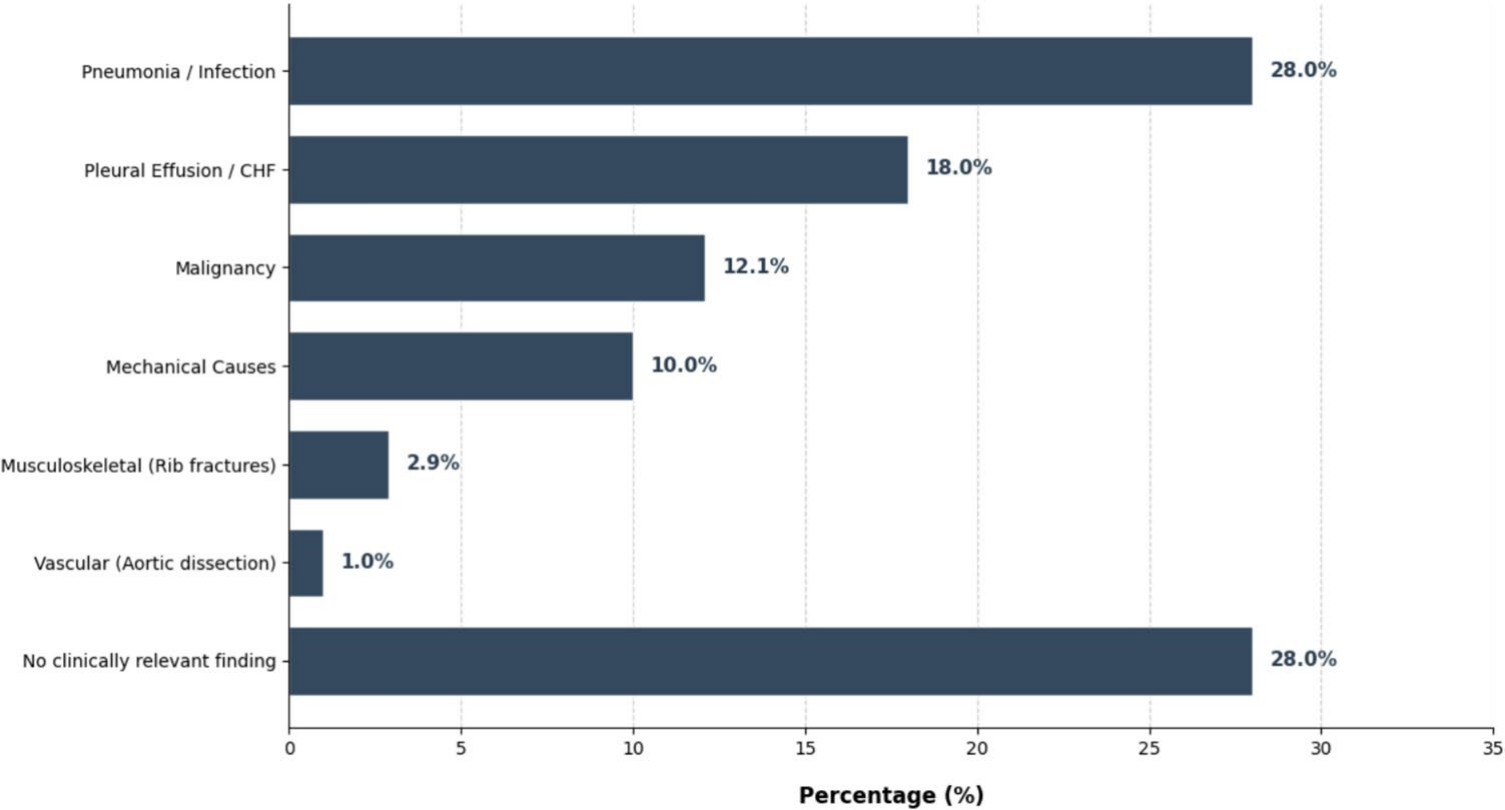
Distribution of alternative diagnoses identified in patients with negative CTPA examinations

CTPA identified clinically meaningful alternative diagnoses in the 521 patients with negative examinations; specifically, in 72.0% (375/521) of these negative scans, the radiologist identified an actionable alternative aetiology for the acute presentation (Table 4, Fig. 3):

- Infectious Mimics (28%): Bronchopneumonia, lobar consolidations, and inflammatory ground-glass opacities were the absolute primary confounders.
- Cardiovascular Mimics (18%): Large pleural effusions, cardiomegaly, and pulmonary edema indicative of decompensated heart failure.
- Oncological Mimics (12%): Incidental discovery of primary lung carcinomas or extensive metastatic disease.
- Mechanical Mimics (10%): Atelectasis, pneumothorax, and severe COPD exacerbations.
- Musculoskeletal/Thoracic Wall Mimics (2.9%): Rib fractures.
- Vascular Mimics (1.0%): Aortic dissection or intramural hematoma.

**Table 4:** D-dimer distribution and alternative thoracic diagnoses in patients undergoing CTPA for suspected pulmonary embolism

Parameter	Category	Value
D-dimer analysis	Median D-dimer level in PE-positive patients	7,840 ng/mL
	Median D-dimer level in PE-negative patients	2,150 ng/mL
	Statistical test	Mann–Whitney U
	p-value	< 0.0001
Alternative diagnoses in PE-negative scans (n = 521)	Pneumonia/infectious consolidation	146 (28.0%)
	Pleural effusion/heart failure	94 (18.0%)
	Malignancy (primary or metastatic)	63 (12.1%)
	Mechanical causes (atelectasis, pneumothorax)	52 (10.0%)
	Musculoskeletal (e.g., rib fractures) Vascular (e.g., aortic dissection) No clinically relevant alternative finding	15 (2.9%) 5 (1.0%) 146 (28.0%)

**Fig. 4:**
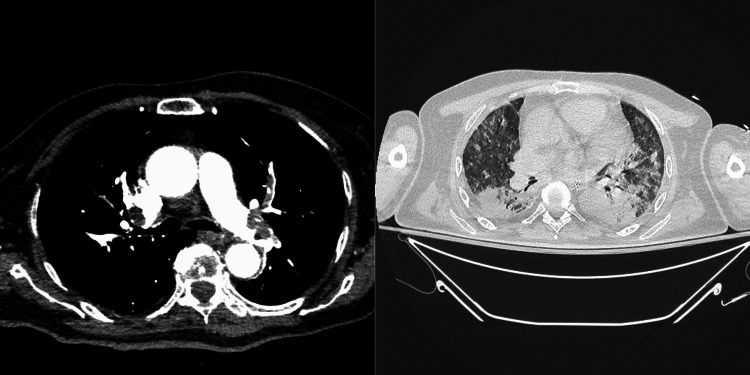
Representative CTPA scans illustrating diagnostic outcomes. (**a**) Positive for pulmonary embolism. Filling defects are visible in the right and left main pulmonary arteries as well as in segmental branches, indicating thromboembolic obstruction. (**b**) Negative for pulmonary embolism, showing multiple bilateral basal pulmonary consolidations, consistent with pneumonia or inflammatory infiltrates

Representative examples of CTPA findings are shown in Fig. 4a–b.

## Discussion

The global diagnostic yield of 20.9% observed in this cohort falls within the 15–25% range recommended by the American College of Radiology and the Royal College of Radiologists [5], indicating appropriate utilization of CTPA in the emergency setting. This confirms that emergency physicians maintain a reasonably accurate pre-test probability assessment, even under the clinical pressure to exclude life-threatening pulmonary embolism. However, we observed a significant drop in diagnostic yield in 2025 (13.9%). While the exact cause is multifactorial, this decline likely reflects increased ED crowding and a lowered clinical threshold for defensive scanning in a post-pandemic environment, pushing the yield below optimal international benchmarks.

A striking finding of our study is the high prevalence of infectious consolidations masquerading as PE. Pathophysiologically, severe pneumonia can mimic PE by causing localized V/Q mismatch (hypoxia), triggering systemic inflammatory responses (leading to D-dimer elevation), and irritating the pleura (chest pain) [7]. This underscores the need for careful symptom assessment and age-adjusted biomarker interpretation.

Age emerged as the strongest determinant of PE prevalence and D-dimer specificity. The Odds Ratio of 2.45 for syncope highlights its role as a "red flag" symptom, warranting immediate imaging regardless of D-dimer values. The Mann–Whitney U analysis confirmed the substantial overlap of D-dimer values between PE-positive and PE-negative patients, demonstrating that in older adults, elevated D-dimer often reflects "thoracic distress" rather than thrombosis. Our findings strongly suggest that adopting age-adjusted D-dimer thresholds could be a potential strategy to reduce unnecessary CTPA scans, thereby acting as an indirect, yet significant, method of radioprotection in the emergency department." [4–10].

Based on our cohort data, we infer that transitioning to age-adjusted D-dimer thresholds (Age × 10 µg/L for patients > 50 years) is a highly effective clinical strategy. A counterfactual projection of our > 70 years cohort indicates that strictly applying the ADJUST-PE criteria [8] could have theoretically safely averted a significant portion of the 42% of false-positive scans, thereby indirectly functioning as a powerful radioprotection measure.

While our study did not measure patient-specific dosimetric data (e.g., CTDIvol or DLP), it is universally accepted that optimization of CTPA utilization is both a diagnostic and a radioprotection imperative [6, 10, 11]. Modern multidetector CT scanners support high-pitch acquisition and iterative reconstruction algorithms, enabling substantial dose reduction [12–14]. Combined with judicious clinical triage, these strategies allow a patient-centered approach to PE evaluation [15].

Overall, these findings reinforce the principle that precision in clinical assessment, biomarker interpretation, and imaging technique is essential to optimize both diagnostic yield and patient safety [16].

The strengths of this study include the relatively large real-world emergency department cohort and the comprehensive characterization of alternative thoracic diagnoses identified on CTPA.

## Limitations

This study acknowledges some methodological limitations. The retrospective design relies entirely on the fidelity of existing radiological and clinical registries, which are subject to documentation bias. Secondly, the absence of standardized clinical probability scoring (such as Wells or Geneva scores) in the electronic records prevented a formal evaluation of strict guideline adherence; therefore, "appropriateness" was inferred primarily through the proxy of diagnostic yield. Furthermore, quantitative D-dimer values were occasionally reported qualitatively, and we did not calculate specific predictive values (Sensitivity/Specificity/PPV/NPV), opting instead to evaluate median biomarker distributions. Finally, as a single-center study, generalizability may be limited, and the lack of cohort-specific radiation dose measurements limits conclusions regarding exact dosimetric savings.

## Conclusion

This study demonstrates that CTPA utilization in the emergency department can achieve an appropriate diagnostic yield when guided by careful clinical assessment. However, diagnostic performance is significantly influenced by patient age and the prevalence of inflammatory pulmonary conditions that mimic embolic disease.

Implementing age-adjusted D-dimer thresholds and structured pre-test probability scoring can substantially reduce unnecessary imaging while maintaining diagnostic safety. Our findings highlight that over two-thirds (72.0%) of negative CTPA examinations provide an alternative diagnosis, reinforcing the role of CTPA as a comprehensive thoracic assessment tool beyond embolism detection [17].

Optimizing CTPA utilization should therefore be considered both a diagnostic and a radioprotection priority, integrating clinical probability assessment, biomarker stratification, and dose-optimized CT protocols to improve both patient safety and healthcare resource utilization.

## Data Availability

The datasets generated and analysed during the current study are available from the corresponding author on reasonable request.

## References

[1] Stein PD et al (2006) Multidetector computed tomography for acute pulmonary embolism (PIOPED II). N Engl J Med 354(22):2317–2327. 10.1056/NEJMoa052367 10.1056/NEJMoa052367 16738268

[2] Becattini C, Agnelli G (2016) Risk stratification and management of acute pulmonary embolism. Hematology Am Soc Hematol Educ Program 2016(1):404–412. 10.1182/asheducation-2016.1.404 27913508 10.1182/asheducation-2016.1.404 PMC6142437

[3] Wells PS et al (2003) Evaluation of D-dimer in the diagnosis of suspected deep-vein thrombosis. N Engl J Med 349(13):1227–1235. 10.1056/NEJMoa023153 10.1056/NEJMoa023153 14507948

[4] Writing Group for the Christopher Study Investigators* (2006) Effectiveness of managing suspected pulmonary embolism using an algorithm combining clinical probability, D-Dimer testing, and computed tomography. JAMA 295(2):172–179. 10.1001/jama.295.2.172 16403929 10.1001/jama.295.2.172

[5] Royal College of Radiologists (2015) BFCR (15)2: clinical audit of the use of CTPA in the diagnosis of PE. RCR, London. https://www.rcr.ac.uk/career-development/audit-quality-improvement/auditlive-radiology-templates/appropriateness-of-usage-of-computed-tomography-pulmonary-angiography-ctpa-investigation-of-suspected-pulmonary-embolism/

[6] Brenner DJ, Hall EJ (2007) Computed tomography — an increasing source of radiation exposure. N Engl J Med 357(22):2277–2284. 10.1056/NEJMra072149 10.1056/NEJMra072149 18046031

[7] van Es J. et al (2013) Clinical impact of findings supporting an alternative diagnosis on CT pulmonary angiography in patients with suspected pulmonary embolism. Chest 144(6):1893-1899. 10.1378/chest.13-0157 10.1378/chest.13-0157 23989896

[8] Righini M et al (2014) Age-adjusted D-dimer cutoff levels to rule out pulmonary embolism: the ADJUST-PE study. JAMA 311(11):1117–1124. 10.1001/jama.2014.2135 10.1001/jama.2014.2135 24643601

[9] Konstantinides SV et al (2020) 2019 ESC Guidelines for the diagnosis and management of acute pulmonary embolism developed in collaboration with the ERS. Eur Heart J 41(4):543–603. 10.1093/eurheartj/ehz405 10.1093/eurheartj/ehz405 31504429

[10] Takahashi EA, Yoon HC (2013) Four-year cumulative radiation exposure in patients undergoing computed tomography angiography for suspected pulmonary embolism. Radiol Res Pract 2013:482403. 10.1155/2013/482403 23984065 10.1155/2013/482403 PMC3745975

[11] Viry A, Vitzthum V, Monnin P et al (2024) Optimization of CT pulmonary angiography for pulmonary embolism using task-based image quality assessment and diagnostic reference levels: a multicentric study. Phys Med 121:103365. 10.1016/j.ejmp.2024.103365 38663347 10.1016/j.ejmp.2024.103365

[12] Schembri GP, Miller AE, Smart R (2010) Radiation dosimetry and safety issues in the investigation of pulmonary embolism. Semin Nucl Med 40(6):442–454. 10.1053/j.semnuclmed.2010.07.007 20920634 10.1053/j.semnuclmed.2010.07.007

[13] Schmid J, Nagy E et al (2022) Diagnosing pulmonary embolism with computed tomography pulmonary angiography: diagnostic accuracy of a reduced scan range. J Thorac Imaging 37(5):323–330. 10.1097/RTI.0000000000000664 35797627 10.1097/RTI.0000000000000664 PMC9394489

[14] Albrecht MH, Bickford MW et al (2017) State-of-the-art pulmonary CT angiography for acute pulmonary embolism. AJR Am J Roentgenol 208(3):495–504. 10.2214/AJR.16.17202 27897042 10.2214/AJR.16.17202

[15] Zieliński D, Dyk et al. (2023) Surgical pulmonary embolectomy: state of the art. Kardiochir Torakochirurgia Pol. 20(2):111-11710.5114/kitp.2023.130019 10.5114/kitp.2023.130019 PMC10410633 37564960

[16] Richman, P.B. et al (2004) Prevalence and Significance of Nonthromboembolic Findings on Chest Computed Tomography Angiography Performed to Rule Out Pulmonary Embolism: A Multicenter Study of 1,025 Emergency Department Patients. 10.1197/j.aem.2003.12.021 15175202

[17] Thomas SE, Weinberg I et al (2024) Diagnosis of pulmonary embolism: A review of evidence-based approaches. J Clin Med 13(13):3722. 10.3390/jcm13133722 10.3390/jcm13133722 PMC11242034 38999289

